# The Example of the American Hospital Association

**Published:** 1916-09-30

**Authors:** 


### The Hospital

The Workers' Newspaper of Administrative Medicine and Institutional Life, Administration, National Insurance and Health.

No. 1582, Vol. LX. SATURDAY, SEPTEMBER 30, 1916. PRICE ONE PENNY.

### THE EXAMPLE OF THE AMERICAN HOSPITAL ASSOCIATION.

The eighteenth annual conference of the American Hospital Association is being held at Philadelphia on September 26-29, as we go to press, and no hospital superintendent can fail to be interested in what promises to be the most important conference yet held by the Association. The first things which will strike anyone who peruses the programme are the amount of organisation which has been expended on the social side of the conference, and the wide range of subjects to be discussed. With the former point, suggestive as it certainly must be to those who have to arrange conferences in this country, we will not deal, but the programme demands careful consideration.

Perhaps the first fact to notice is one of method. Some special sessions have been arranged for problems concerning the smaller hospitals, and members of the British Hospitals Association will note this point because, at the conferences which have been held by our own Association in this country, the feeling has been expressed that while it was certainly desirable that the superintendents of all the hospitals should be members of the Association, yet many of the questions to be discussed at the annual meetings have suffered in the past from the interposition of speakers who forgot that the relative bigness or smallness of their own institution prevented them from usefully discussing some paper or point which could be elucidated only by representatives of hospitals that were of a size to have practical experience of it. The men from the small hospitals could contribute little to a discussion affecting great hospitals only, and *vice versa*. Yet so long as one session only was held, representatives either had to attend or feel that they were losing an opportunity for a meeting, or, worst of all, to join in a discussion of points that lay outside their purview. A special session for small hospitals solves these difficulties. The holding of such sessions, moreover, seems to justify a suggestion, now under discussion, which has not been found workable in this country—the suggestion, namely, that membership of the American Hospital Association should not be confined to superintendents only, but be open to all actively engaged in hospital work. In England, of course, we have two Associations, one for senior, the other for junior men, nor do we see how they can be usefully combined in one Association unless some division be made, and perhaps a system of separate sessions for large and small hospitals is as convenient as any.

When we pass from these and other questions concerning the domestic politics of the American Hospital Association to the papers on general matters, the list is varied and important. Those in which members of the British Hospitals Association will be most interested include one on "Medical Organisation and Medical Education," by Dr. Charles Young, superintendent of the Presbyterian Hospital, New York. The point of interest is that at this hospital a hard-and-fast line of demarcation exists between the respective duties and activities of the lay and medical staffs. The question whether outside physicians should be allowed to bring in paying patients and have equal privileges with those of the hospital staff forms the subject of another paper, and is of special interest to us from the fact that the growth of pay-wards in this country will shortly bring forward similar problems to those that have now reached the stage of controversy in America. "Venereal Clinics and their Proper Lines of Development," on which Dr. W. F. Snow, secretary of the American Social Hygiene Association, will read a paper, has a topical interest for ourselves which needs no further emphasis. Dr. Ancker's report for the Committee on Hospital Construction will be studied with interest owing to the lengthy experience which he has had of building hospitals. "The Hospital Dietary," "Practical Methods of

Preventing the Spread of Infection," "Autopsies," and the problems they create, "Dental Clinics in General Hospitals," "The Cost of Accounting," are dealt with in other papers, and those which we have mentioned by no means exhaust the list.

What is the moral to be drawn from it for the British Hospitals Association? On the interest of the programme for individual superintendents we need not dwell; they can be relied on to draw whatever information they want from it. But we suggest that the British Hospitals Association is offered much to think of by this programme, and that individual superintendents would do well to study the conference not merely from their own point of view, but as members of the British Hospitals Association. In England, it can hardly be denied, some want of cohesion exists. The big hospital in London, at least, tends to depend upon the influence of its chairman or treasurer, whose status in public life or in the House of Lords is felt sufficient to press the interests of the institution which he represents at any moment when they may be felt to be endangered. The result is that each London hospital follows, often as a matter of policy, its own aims as an independent unit, and that only some new and far-reaching legislation, like the Insurance Act for instance, succeeds in bringing some greater measure of cohesion into the hospital world. In the provinces, on the whole, there is more solidarity. But even here such real co-operation as exists takes place mostly behind the scenes, and through the letter-boxes of individual superintendents. We cannot leave this important conference, in short, without asking ourselves how far our habit of isolation, especially in London, is a wise one, and how far we ought to adopt the American plan of more developed organisation.

